# Quantifying Decoherence via Increases in Classicality

**DOI:** 10.3390/e23121594

**Published:** 2021-11-28

**Authors:** Shuangshuang Fu, Shunlong Luo

**Affiliations:** 1 School of Mathematics and Physics, University of Science and Technology Beijing, Beijing 100083, China; shuangshuang.fu@ustb.edu.cn; 2 Academy of Mathematics and Systems Science, Chinese Academy of Sciences, Beijing 100190, China; 3 School of Mathematical Sciences, University of Chinese Academy of Sciences, Beijing 100049, China

**Keywords:** decoherence, classicality, channel, open system, interference

## Abstract

As a direct consequence of the interplay between the superposition principle of quantum mechanics and the dynamics of open systems, decoherence is a recurring theme in both foundational and experimental exploration of the quantum realm. Decoherence is intimately related to information leakage of open systems and is usually formulated in the setup of “system + environment” as information acquisition of the environment (observer) from the system. As such, it has been mainly characterized via correlations (e.g., quantum mutual information, discord, and entanglement). Decoherence combined with redundant proliferation of the system information to multiple fragments of environment yields the scenario of quantum Darwinism, which is now a widely recognized framework for addressing the quantum-to-classical transition: the emergence of the apparent classical reality from the enigmatic quantum substrate. Despite the half-century development of the notion of decoherence, there are still many aspects awaiting investigations. In this work, we introduce two quantifiers of classicality via the Jordan product and uncertainty, respectively, and then employ them to quantify decoherence from an information-theoretic perspective. As a comparison, we also study the influence of the system on the environment.

## 1. Introduction

A fundamental hallmark of quantum mechanics is the superposition principle [1,2], which leads naturally to coherence and interference [3]. Although reduced coherence (e.g., Landau’s study of wave damping [4]) and suppression of interference (e.g., Mott’s analysis of α-particle tracking [5]) have featured early studies ever since the beginning of quantum mechanics, the modern conceptualization of the idea of decoherence as a subject in its own right started only in the 1970s, as initiated by Zeh and Zurek [6,7,8,9,10]. The influential and seminal work of Zurek has led further to the development of quantum Darwinism. Nowadays, decoherence has been a subject of many studies after surprising neglect at the initial stage and has gained increasingly importance with the deep investigations of quantum measurement and the emergence of quantum information.

Decoherence provides an elegant mechanism for exploring the boundary between classical and quantum behaviors and imposes technological limits for quantum devices. An ultimate goal of quantum information science is to construct quantum computers, which are notoriously fragile and prone to decoherence, and they call for combating decoherence for quantum information processing [11]. Decoherence also plays a significant role in designing error correction codes, as the notion of decoherence-free schemes (subspace) indicates. Of course, decoherence actually has many more applications to quantum science than to quantum computing per se.

Formally, decoherence usually refers to the decay of the off-diagonal entries of the system density matrix (in the basis of the pointer observable) caused by evolution of the combined “system + environment”. Alternatively, it is also characterized as the establishing of correlations between the system and the environment, which causes the system to behave in a classical manner. In this context, the environment effectively measures (monitors) the system. This relational scenario indicates that decoherence is a relative concept and has to be characterized with respect to a reference basis and an environment. In a more pedantic and rigorous fashion, when we talk about decoherence, we should bear in mind (explicitly/implicitly) three ingredients:

(1) Decoherence of which (state)?
(2) Decoherence relative to which (basis, or more generally, channel)?
(3) Decoherence caused by which (environment or observation)?

Decoherence is intimately related to a range of fundamental quantum issues such as the measurement problem [12,13,14,15,16,17], entanglement and nonlocality [18], irreversibility (the arrow of time) [19], and the quantum-to-classical transition [8,9,10,12,13,14,15,16,17,18]. The later seeks an explanation of the apparent transition from the quantum realm to the classical realm (i.e., the emergence of a classical objective reality from the quantum substrate) as described by quantum Darwinism [20,21,22,23,24,25,26,27,28,29,30]. Decoherence serves as a natural arena for the interplay among wave–particle duality [31,32,33,34,35,36], wave-packet collapse [8,9,37,38], information transferring [39,40,41], state broadcasting [42,43,44,45,46,47], and quantum correlations (quantum discord) [48,49,50,51,52,53,54]. Decoherence is also employed in the theory of decoherent histories (consistent histories) approach to quantum mechanics [55,56,57,58,59].

Coherence and decoherence are complementary to each other, or, phrased alternatively, they are the two sides of the same coin: decoherence is just loss of coherence. Coherence arises from the superposition principle and means that a state is in superposition of several states operating together in a coherent way, and decoherence means the loss of this behavior or, more precisely, the loss of definite phase relation between the constituent states for the superposition and thus results in classical mixture of states. Coherence and decoherence play a pivotal role in studying the theoretical issue of the quantum-to-classical transition and in investigating the practical issue concerning physical realization of quantum information processing.

Decoherence is intimately related to loss of quantumness or, put alternatively, an increase in classicality. The quantumness of states and ensembles were studied from various perspectives [60,61,62,63,64,65,66,67,68,69,70,71,72]. In particular, the use of non-commutativity as a quantumness witness for a single system was proposed and experimentally confirmed in Refs. [64,65,66,67]. An explicit relation between the Jordan product of operators and quantumness was discussed in [70]. A method for measuring quantumness in interferometric setups was presented in [72].

In this work, motivated by previous studies, and following quantitative investigations of coherence and superposition [73,74,75,76,77,78,79,80,81,82,83,84], we aimed at quantifying decoherence induced by the environment, which may be helpful for quantitatively characterizing certain features of the quantum-to-classical transition and quantum Darwinism.

The remainder of the article is arranged as follows. In Section 2, we present some preliminary results. In particular, we introduce two quantifiers of classicality in terms of operator anti-commutators (symmetric and the Jordan product of operators) and a modified variance in a general setup, which, apart from their use in quantifying decoherence, may be of independent interest. Two inequalities for monotonicity of classicality are established. In Section 3, we introduce two quantifiers of decoherence by exploiting the monotonicity of classicality and reveal their basic features. In Section 4, we discuss the influence on the environment caused by the system, which stands in contrast to decoherence of the system induced by the environment. In Section 5, we illustrate the quantifiers of decoherence in a two-path interferometer. Finally, we summarize the results and present some discussions in Section 6. For simplicity, we consider only finite dimensional systems, although it seems that many results can be readily extended to infinite dimensional cases.

## 2. Preliminaries

In this section, we consider a general setup of state–channel interaction and discuss two quantifiers of classicality, which will be used to quantify decoherence in the next section. The first quantifier involves the Jordan product of operators and is intimately connected to the Wigner–Yanase skew information [84]. The second is defined via variance of a state, which stands in some sense dual to the conventional variance of an observable [85,86].

Decoherence is sometimes also called dephasing, dynamical decoherence, or environment-induced decoherence. Here, we emphasize the role of the environment in inducing decoherence. In the conventional approach, decoherence is often read from the off-diagonal entries of the reduced system density matrix after it interacts with the environment, which provide a rather complete picture of the effect of decoherence. However, since all these off-diagonal entries still constitute a matrix with vanishing diagonals, one may be interested in summarizing decoherence by a single numerical quantity, just like although a quantum state provides a complete description of a system, one still seeks certain functionals of the state, such as the von Neumann entropy and purity, to capture some essential features of the state.

Decoherence is usually studied in the context of an open system, which is coupled with the environment. When we focus on the system and ignore the environment, the dynamics of a state ρ of a *d*-dimensional quantum system is mathematically described by a quantum channel (here we only consider the case with the same input and output system) in the Kraus representation form [11,87]

(1) $$K\left(\rho \right)=\sum_{i}K_{i}\rho K_{i}^{†},$$

where $K_{i}$ are the Kraus operators (effects) satisfying $\sum_{i}K_{i}^{†}K_{i}=1$ (the identity operator), which ensures trace-preservation of the channel K. If moreover $\sum_{i}K_{i}K_{i}^{†}=1,$ then the channel is called unital: it leaves the maximally mixed state (proportional to the identity operator) invariant. We remark that in Equation (1) if we replace ρ by any operator *X*, the above operation still makes sense as a map. This channel will serve as a reference channel when we talk about decoherence (with respect to K) and actually may be regarded as a generalization of an orthonormal basis, which induces a von Neumann measurement. Consequently, a general notion of decoherence goes beyond that based on an orthonormal basis (pointer observable).

### 2.1. Classicality in Terms of the Jordan Product

In order to establish notation and to motivate our approach to decoherence, we first recall certain information-theoretical features of state–channel interaction [84]. For the channel determined by Equation (1), let

(2) $$J\left(\rho ,K\right)=\frac{1}{2}\sum_{i}\mathrm{tr}\left(\left\{\sqrt{\rho },K_{i}\right\}{\left\{\sqrt{\rho },K_{i}\right\}}^{†}\right),$$

which will be interpreted as a kind of measure of classicality for the state–channel interaction (or as the classicality of the state with respect to the channel), as will be elucidated later. Here,

{X,Y}=XY+YX

denotes the anti-commutator (the Jordan product or the symmetric product) of operators *X* and *Y* acting on the system Hilbert space. This commutative product indicates certain features of classicality. Indeed, decoherence is intimately related to the appearance or increasing of classicality, and the symmetric Jordan product, as a commutative operation, is also intimately related to classicality. Consequently, it is plausible and reasonable that decoherence may be quantified via the Jordan product of states and observables, just like that quantumness of states can be characterized via the Jordan product of observables [70]. After simple manipulation, we have

(3) $$J\left(\rho ,K\right)=\frac{1}{2}\mathrm{tr}\left(K\left(\rho \right)+K^{†}\left(\rho \right)+2\sqrt{\rho }K^{†}\left(\sqrt{\rho }\right)\right),$$

where

$$K^{†}\left(X\right)=\sum_{i}K_{i}^{†}XK_{i}$$

is the dual channel of K. We notice that $\mathrm{tr}\left(\sqrt{\rho }K^{†}\left(\sqrt{\rho }\right)\right)=\mathrm{tr}\left(K^{†}\left(\sqrt{\rho }\right)\sqrt{\rho }\right)=\mathrm{tr}\left(\sqrt{\rho }K\left(\sqrt{\rho }\right)\right).$

It is well known that the Kraus operators for the operator-sum representation in Equation (1) are not unique, and the question arises as to whether the defining Equation (2) for the classicality quantifier J(ρ,K) is well defined. Indeed, the Kraus operators $K_{i}$ for different representations of the channel K are related by unitary transformations (the unitary freedom in the operator-sum representation of a channel) [11], and it can be readily shown that J(ρ,K) is independent of the choice of the Kraus operators and is thus unambiguously defined.

For comparison, we also introduce

(4) $$I\left(\rho ,K\right)=\frac{1}{2}\sum_{i}\mathrm{tr}\left(\left[\sqrt{\rho },K_{i}\right]{\left[\sqrt{\rho },K_{i}\right]}^{†}\right),$$

where

[X,Y]=XY−YX

denotes the commutator (the Lie product or the anti-symmetric product) of the operators *X* and *Y*. Clearly,

(5) $$I\left(\rho ,K\right)=\frac{1}{2}\mathrm{tr}\left(K\left(\rho \right)+K^{†}\left(\rho \right)-2\sqrt{\rho }K^{†}\left(\sqrt{\rho }\right)\right).$$

It is remarkable that if $K_{i}$ is a Hermitian operator, then the summand

$$\frac{1}{2}\mathrm{tr}\left(\left[\sqrt{\rho },K_{i}\right]{\left[\sqrt{\rho },K_{i}\right]}^{†}\right)=-\frac{1}{2}\mathrm{tr}\left({\left[\sqrt{\rho },K_{i}\right]}^{2}\right)$$

in Equation (4) is precisely the celebrated Wigner–Yanase skew information

(6) $$I\left(\rho ,K_{i}\right)=-\frac{1}{2}\mathrm{tr}\left({\left[\sqrt{\rho },K_{i}\right]}^{2}\right)$$

of ρ (with $K_{i}$ serving as a conserved observable) [88], which is now playing an increasingly interesting and important role in quantum theory [89,90,91,92,93,94,95,96,97]. In particular, the Wigner–Yanase skew information is monotone in the sense that [98,99]

(7) I(Φ(ρ),K)≤I(ρ,K)

for any channel Φ that does not disturb the observable *K* (i.e., $\Phi^{†}\left(K\right)=K,\Phi^{†}\left(K^{2}\right)=K^{2}$). This will be used to establish Proposition 1.

It is well recognized that the Wigner–Yanase skew information is a particular version of quantum Fisher information [90], which is quite different from the quantum (von Neumann) entropy. Actually, the original motivation for Wigner and Yanase introducing the skew information was to seek an alternative quantity for quantifying information contents of quantum states in the presence of conserved observables.

By Equations (3) and (5) and the fact that trρ=1, if the channel K is unital in the sense that K(1)=1 (equivalently, $\sum_{i}K_{i}K_{i}^{†}=1$), then

(8) J(ρ,K)+I(ρ,K)=2,

which shows that J(ρ,K) (involving the symmetric Jordan product) and I(ρ,K) (involving the anti-symmetric Lie product) are complementary to each other. Moreover,

2≥J(ρ,K)≥1≥I(ρ,K)≥0.

We first list some basic properties of J(ρ,K).

(a) 1≤J(ρ,K)≤2. Moreover, J(ρ,K)=2 if and only if $[\sqrt{\rho },K_{i}]=0$ for all *i*.
(b) J(ρ,K) is concave in ρ.
(c) J(ρ,K) is covariant in the sense that
  $$J\left(U\rho U^{†},K\right)=J\left(\rho ,U^{†}KU\right)$$
  for any unitary operator *U* on the system Hilbert space. Here $U^{†}KU\left(\rho \right)=\sum_{i}\left(U^{†}K_{i}U\right)$$\rho {\left(U^{†}K_{i}U\right)}^{†}.$

Item (a) is apparent from the definition, and item (b) follows from Equation (3) and the celebrated Lieb concavity [100,101], which states that the functional $\mathrm{tr}(\rho^{s}X\rho^{1-s}X^{†})$ is concave in the state ρ for any s∈(0,1) and any operator *X*. Here we only used the case s=1/2. Item (c) can be readily checked.

Recall that the dual of the channel $\Phi \left(\rho \right)=\sum_{i}K_{i}\rho K_{i}^{†}$ is defined as $\Phi^{†}\left(X\right)=\sum_{i}K_{i}^{†}XK_{i}$ for any operator *X*. The following monotonicity of J(ρ,K) under certain channels Φ plays a crucial role in our approach to decoherence.

**Proposition** **1.** *Let* Φ *be a unital channel that does not disturb the Kraus operators of the reference channel K defined by Equation (1) in the sense that $\Phi^{†}\left(K_{i}\right)=K_{i}$ and $\Phi^{†}\left(K_{i}K_{i}^{†}\right)=K_{i}K_{i}^{†}$ for all i, then*

(9) J(ρ,K)≤J(Φ(ρ),K).

Noting the complementarity relation (8), the above monotonicity may be directly derived from the corresponding property of the Wigner–Yanase skew information, as described in Equation (7).

In view of the above increasing behavior (under certain channels) and the properties specified by items (a)–(c), we may interpret J(ρ,K) as a quantifier of classicality of the state ρ (with reference to the channel K). Indeed, a reasonable measure of classicality should be concave in the state ρ (classical mixing of states should not decrease classicality on average), which is in accordance with item (b). Operations on the state that leave the reference channel undisturbed also should not decrease classicality, which is guaranteed by inequality (9). In contrast, I(ρ,K) may be regarded as a quantity of coherence or quantumness of ρ (with reference to K). This is consistent with Equation (8), which may be regarded as an information-theoretic manifestation of the Bohr complementarity from the perspective of the asymmetry–symmetry trade-off [84]: I(ρ,K) characterizes the asymmetry (of ρ with respect to K) and can be related to the path feature in an interferometric setup, while J(ρ,K) characterizes the symmetry (of ρ with respect to K) and can be related to fringe visibility.

### 2.2. Classicality in Terms of Uncertainty

Although the quantity J(ρ,K) has nice information-theoretic features, it involves the square root of a state and thus may be difficult to calculate. For simplicity and comparison, we also introduce an alternative measure of classicality without the square root, which is directly based on a modification of the ubiquitous notion of variance (uncertainty).

Recall that any state, as a Hermitian operator, can also be formally regarded as an observable, and thus one may consider its variance with respect to another state (or, more generally, any operator) [85,86]. Following this consideration, we introduce the variance of a state ρ in a channel K defined by Equation (1) as

(10) $$V_{K}\left(\rho \right)=\sum_{i}V_{K_{i}}\left(\rho \right),$$

where

(11) $$V_{K}\left(\rho \right)=\mathrm{tr}\left({\left(\rho -\mathrm{tr}\left(\rho K^{†}K\right)\right)}^{2}K^{†}K\right)$$

is the generalized variance of ρ (considered as an observable) in *K* (not necessarily a Hermitian operator). It turns out that

(12) $$V_{K}\left(\rho \right)=\mathrm{tr}\left(\rho^{2}K^{†}K\right)-{\left(\mathrm{tr}(\rho K^{†}K)\right)}^{2}\left(2-\mathrm{tr}(K^{†}K)\right)$$

and in particular, if $\mathrm{tr}(K^{†}K)=1,$ then

$$V_{K}\left(\rho \right)=\mathrm{tr}\left(\rho^{2}K^{†}K\right)-{\left(\mathrm{tr}(\rho K^{†}K)\right)}^{2}.$$

In a *d*-dimensional system, the variance of ρ in K is upper bounded as

(13) $$\begin{array}{cc} V_{K}\left(\rho \right) & =\mathrm{tr}\left(\rho^{2}\right)-\sum_{i}{\left(\mathrm{tr}(\rho K_{i}^{†}K_{i})\right)}^{2}\left(2-\mathrm{tr}(K_{i}^{†}K_{i})\right)\\ \; & \leq \mathrm{tr}\left(\rho^{2}\right)-\sum_{i}{\left(\mathrm{tr}(\rho K_{i}^{†}K_{i})\right)}^{2}\left(2-d\right)\\ \; & \leq 1+\left(d-2\right)\sum_{i}{\left(\mathrm{tr}(\rho K_{i}^{†}K_{i})\right)}^{2}\\ \; & \leq 1+(d-2)\\ \; & =d-1. \end{array}$$

Furthermore, if $\mathrm{tr}(K_{i}^{†}K_{i})=1$ for all *i*, then we actually have

(14) $$V_{K}\left(\rho \right)=\mathrm{tr}\left(\rho^{2}\right)-\sum_{i}(\mathrm{tr}{\left(\rho K_{i}^{†}K_{i})\right)}^{2}\leq 1.$$

For some applications and intuitions of the above quantities, see Refs. [85,86]. In Equation (10), we have put *K* in the subscript, i.e., with the notation $V_{K}\left(\rho \right)$ rather than V(ρ,K); we are emphasizing that the above variance is quite different from the conventional variance $V\left(\rho ,K\right)=\mathrm{tr}(\rho {\left(K-\mathrm{tr}\rho K\right)}^{2})$ of the observable *K* (in the state ρ). Indeed, $V_{K}\left(\rho \right)$ is convex in ρ, while V(ρ,K) is concave in ρ.

To introduce an alternative quantifier of classicality, noting that $V_{K}\left(\rho \right)\leq d-1$, we define

(15) $$C\left(\rho ,K\right)=d-1-V_{K}\left(\rho \right)=S_{2}\left(\rho \right)+\sum_{i}{\left(\mathrm{tr}(\rho K_{i}^{†}K_{i})\right)}^{2}\left(2-\mathrm{tr}(K_{i}^{†}K_{i})\right)+d-2,$$

where

$$S_{2}\left(\rho \right)=1-\mathrm{tr}\left(\rho^{2}\right)$$

is the Tsallis 2-entropy. The quantity C(ρ,K) has the following properties.

(a)
0≤C(ρ,K)≤d−1.

(b) C(ρ,K) is concave in ρ.
(c) C(ρ,K) is covariant in the sense that
  $$C\left(U\rho U^{†},K\right)=C\left(\rho ,U^{†}KU\right)$$
  for any unitary operator *U* on the system Hilbert space. Here the channel $U^{†}KU$ is defined as $U^{†}KU\left(\rho \right)=\sum_{i}\left(U^{†}K_{i}U\right)\rho {\left(U^{†}K_{i}U\right)}^{†}.$

Similar to J(ρ,K), we have the following monotonicity property.

**Proposition** **2.** *Let* Φ *be a unital channel that does not disturb the Kraus operators of the reference channel K defined by Equation (1) in the sense that $\Phi^{†}\left(K_{i}^{†}K_{i}\right)=K_{i}^{†}K_{i}$ for all i, then*

(16) C(ρ,K)≤C(Φ(ρ),K).

In order to prove the above statement and to characterize the effect of the channel Φ, we first recall the notion of majorization for vectors [102,103,104]. For any real vector $x=\left(x_{1},x_{2},\cdots ,x_{d}\right)\in R^{d},$ let $x^{↓}=\left(x_{1}^{↓},x_{2}^{↓},\cdots ,x_{d}^{↓}\right)$ be the vector obtained by rearranging the components of x in a non-increasing order. The weak majorization relation $x{≼}_{w}y$ (i.e., x is weakly majorized by y, or y weakly majorizes x) means that [103]

$$\begin{array}{c} \sum_{i=1}^{k}x_{i}^{↓}\leq \sum_{i=1}^{k}y_{i}^{↓},\;k=1,2,\cdots ,d. \end{array}$$

If furthermore $\sum_{i=1}^{d}x_{i}^{↓}=\sum_{i=1}^{d}y_{i}^{↓}$ (which is always satisfied for probability vectors), then it is said that x is majorized by y, denoted as x≼y. Intuitively, x≼y means that x is more chaotic (more flat, more uniform, more mixed, and more spread out) than y. For example,

$$\left(\frac{1}{d},\frac{1}{d},\cdots ,\frac{1}{d}\right)≼\left(x_{1},x_{2},\cdots ,x_{d}\right)≼\left(1,0,\cdots ,0\right)$$

for any $x_{i}\geq 0,\sum_{i=1}^{d}x_{i}=1.$ It is well known that x≼y if and only if x=My for some doubly stochastic matrix *M* (i.e., square matrix with non-negative entries and all row and column sums equal to 1) [102]. We will be only concerned with probability vectors arising from eigenvalues of a quantum state (density matrix).

Now, by the condition $\Phi^{†}\left(K_{i}^{†}K_{i}\right)=K_{i}^{†}K_{i}$ we obtain

$$\mathrm{tr}\left(\Phi \left(\rho \right)K_{i}^{†}K_{i}\right)=\mathrm{tr}\left(\rho \Phi^{†}\left(K_{i}^{†}K_{i}\right)\right)=\mathrm{tr}\left(\rho K_{i}^{†}K_{i}\right).$$

Consequently, under the above condition, inequality (16) is equivalent to

$$\mathrm{tr}\left(\Phi {\left(\rho \right)}^{2}\right)\leq \mathrm{tr}\left(\rho^{2}\right),$$

which is true for any unital channel.

## 3. Quantifying Decoherence of System Induced by Environment

With the above preparation, which is a rather general setup, we now proceed to quantify decoherence of system induced by environment. In order to obtain more concrete and explicit results, we have to specify the system-environment coupling. For simplicity, we study the important case when the reference channel K is induced by a von Neumann measurement $\Pi =\{\Pi_{i}=|i\rangle \langle i|:i=1,2,\cdots ,d\}$ in a *d*-dimensional system with {|i〉:i=1,2,⋯,d} being an orthonormal basis of the system Hilbert space. In this case, we write the corresponding reference channel as

$$\Pi \left(\rho \right)=\sum_{i=1}^{d}\Pi_{i}\rho \Pi_{i}=\sum_{i=1}^{d}\langle i|\rho |i\rangle |i\rangle \langle i|.$$

By specifying K to Π and noting Equation (3), we have

(17) $$J\left(\rho ,\Pi \right)=1+\mathrm{tr}\left(\sqrt{\rho }\Pi^{†}\left(\sqrt{\rho }\right)\right)=1+\sum_{i=1}^{d}\langle i|\sqrt{\rho }{|i\rangle }^{2}.$$

Consider a quantum system with a *d*-dimensional Hilbert space $H_{S}$, which interacts with an environment consisting of *d* parts described by the Hilbert space $H_{E}=H_{E_{1}}⊗H_{E_{2}}⊗\cdots ⊗H_{E_{d}}$ through the controlled unitary operation

(18) $$\Pi_{U}=\Pi_{1}⊗U_{E_{1}}+\Pi_{2}⊗U_{E_{2}}+\cdots +\Pi_{d}⊗U_{E_{d}}$$

on the combined system

$$H_{S}⊗H_{E}=H_{S}⊗\left(H_{E_{1}}⊗H_{E_{2}}⊗\cdots ⊗H_{E_{d}}\right).$$

**Figure 1:**
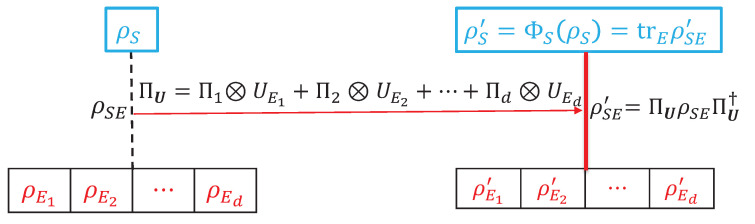
Schematic illustration of decoherence of the system (initially in the state $\rho_{S}$) induced by the environment consisting of *d* sub-environments (initially in the state $\rho_{E}=\rho_{E_{1}}⊗\rho_{E_{2}}⊗\cdots ⊗\rho_{E_{d}}).$ The combined initial system–environment state is $\rho_{SE}=\rho_{S}⊗\rho_{E}.$ The system and environment is coupled via the combined unitary operator $\Pi_{U}=\sum_{i=1}^{d}\Pi_{i}⊗U_{E_{i}},$ and the final combined system–environment state is $\rho_{SE}^{\prime }=\Pi_{U}\rho_{SE}\Pi_{U}^{†}$ with final system state $\rho_{S}^{\prime }=\mathrm{tr}\rho_{SE}^{\prime }.$ The decoherence of $\rho_{S}$ (with respect to Π) induced by the environment is quantified by $D\left(\rho_{S}|\Pi ,U\right)=J\left(\rho_{S}^{\prime },\Pi \right)-J\left(\rho_{S},\Pi \right)$ (see Equation (27)) and $F\left(\rho_{S}|\Pi ,U\right)=C\left(\rho_{S}^{\prime },\Pi \right)-C\left(\rho_{S},\Pi \right)$ (see Equation (31)), both of which may be interpreted as increases in classicality of the system caused by the environment.

Here $U=\{U_{E_{i}}:i=1,2,\cdots ,d\}$ denotes the collection of unitary operators on the various sub-environments, with $U_{E_{i}}$ acting only on $H_{E_{i}}$. This scenario is schematically depicted in Figure 1, and will be further discussed in the next section. In this context, one may wonder why the dimension of the quantum system is the same as the number of environments. This arises naturally in a multi-path interferometer, in which we are only concerned with the path degree of freedoms, and thus the associated system Hilbert space is spanned by the path basis. Consequently, the system is *d*-dimensional when we have *d* paths. In order to study the decoherence of the system (consisting of the *d* paths as an orthonormal basis), we attach a detector to each path, and thus we have *d* sub-environments (corresponding to the *d* detectors).

The initial combined system–environment state is

$$\rho_{SE}=\rho_{S}⊗\rho_{E},$$

where $\rho_{S}$ is the initial system state, and $\rho_{E}=\rho_{E_{1}}⊗\rho_{E_{2}}⊗\cdots ⊗\rho_{E_{d}}$ is the initial environment state. When expressed as a density matrix with respect to the orthonormal basis {|i〉:i=1,2,⋯,d}, the initial system state $\rho_{S}$ has the matrix form

(19) $$\rho_{S}=\left(\langle i|\rho_{S}|j\rangle \right)=\left(\begin{array}{cccc} \langle 1|\rho_{S}|1\rangle & \langle 1|\rho_{S}|2\rangle & \cdots & \langle 1|\rho_{S}|d\rangle \\ \langle 2|\rho_{S}|1\rangle & \langle 2|\rho_{S}|2\rangle & \cdots & \langle 2|\rho_{S}|d\rangle \\ \vdots & \vdots & \ddots & \vdots \\ \langle d|\rho_{S}|1\rangle & \langle d|\rho_{S}|2\rangle & \cdots & \langle d|\rho_{S}|d\rangle \end{array}\right).$$

After the system–environment interaction through the unitary operation (18), the final state of the combined system is

(20) $$\begin{array}{cc} \rho_{SE}^{\prime } & =\Pi_{U}\rho_{SE}\Pi_{U}^{†}\\ \; & =\left(\Pi_{1}⊗U_{E_{1}}+\cdots +\Pi_{d}⊗U_{E_{d}}\right)\left(\rho_{S}⊗\rho_{E}\right){\left(\Pi_{1}⊗U_{E_{1}}+\cdots +\Pi_{d}⊗U_{E_{d}}\right)}^{†}\\ \; & =\sum_{i,j=1}^{d}\left(\Pi_{i}\rho_{S}\Pi_{j}\right)⊗\left(U_{E_{i}}\rho_{E}U_{E_{j}}^{†}\right). \end{array}$$

Here we emphasize that $U_{E_{i}}$ acts only nontrivially on $H_{E_{i}}.$ From the above expression, we obtain the final (reduced) system state

(21) $$\rho_{S}^{\prime }={\mathrm{tr}}_{E}\rho_{SE}^{\prime }=\sum_{i,j=1}^{d}\mathrm{tr}\left(U_{E_{i}}\rho_{E}U_{E_{j}}^{†}\right)\cdot \Pi_{i}\rho_{S}\Pi_{j}$$

after the interaction, which can be represented as the d×d matrix (noting that $\Pi_{i}=\left|i\rangle \langle i\right|$)

(22) $$\rho_{S}^{\prime }=\left(\langle i|\rho_{S}|j\rangle \omega_{ij}\right)=\left(\begin{array}{cccc} \langle 1|\rho_{S}|1\rangle & \langle 1|\rho_{S}|2\rangle \omega_{12} & \cdots & \langle 1|\rho_{S}|d\rangle \omega_{1d}\\ \langle 2|\rho_{S}|1\rangle \omega_{21} & \langle 2|\rho_{S}|2\rangle & \cdots & \langle 2|\rho_{S}|d\rangle \omega_{2d}\\ \vdots & \vdots & \ddots & \vdots \\ \langle d|\rho_{S}|1\rangle \omega_{d1} & \langle d|\rho_{S}|2\rangle \omega_{d2} & \cdots & \langle d|\rho_{S}|d\rangle \end{array}\right)=\rho_{S}\circ \Omega$$

with respect to the orthonormal basis {|i〉:i=1,2,⋯,d}. Here the symbol ∘ denotes the Hadamard product (also called the Schur product or the entry-wise product) of matrices defined as $\left(a_{ij}\right)\circ \left(b_{ij}\right)=\left(a_{ij}b_{ij}\right),$ and $\Omega =(\omega_{ij})$ with

$$\omega_{ij}=\mathrm{tr}\left(U_{E_{i}}\rho_{E}U_{E_{j}}^{†}\right)$$

being a correlation matrix, i.e., a non-negative definite matrix with diagonal entries all equal to 1. By casting $\omega_{ij}$ as

$$\omega_{ij}=\mathrm{tr}\left(U_{E_{i}}\rho_{E}U_{E_{j}}^{†}\right)=\mathrm{tr}\left(X_{i}X_{j}^{†}\right),$$

with $X_{i}=U_{E_{i}}\sqrt{\rho_{E}},$ we readily see that the matrix $\Omega =(\omega_{ij})$ is a Gram matrix of the family of operators $\left\{X_{i}:i=1,2,\cdots ,d\right\}$ as vectors in the Hilbert space consisting of operators (with the Hilbert–Schmidt product) acting on the environment. Moreover,

$$\omega_{ij}=\left\{\begin{array}{c} 1,\;\;i=j\\ \omega_{i}\omega_{j}^{*},\;i\neq j \end{array}\right.$$

with $\omega_{i}=\mathrm{tr}\left(U_{E_{i}}\rho_{E_{i}}\right),$ and $\omega_{j}^{*}$ denotes the complex conjugation of the complex number $\omega_{j}.$ Since Ω is a non-negative definite matrix, it has a square root, which may be symbolically expressed as

(23) $$\sqrt{\Omega }=\left(\alpha_{ij}\right)=\left(\begin{array}{c} \langle \alpha_{1}|\\ \langle \alpha_{2}|\\ \vdots \\ \langle \alpha_{d}| \end{array}\right)=(|\alpha_{1}\rangle ,|\alpha_{2}\rangle ,\cdots ,|\alpha_{d}\rangle ),$$

where the bra $\langle \alpha_{i}|=\sum_{j=1}^{d}\alpha_{ij}\langle j|$ is identified with the row vector $(\alpha_{i1},\alpha_{i2},\cdots ,\alpha_{id}),$ while the corresponding adjoint vector (ket) $|\alpha_{i}\rangle =\sum_{j=1}^{d}\alpha_{ij}^{*}|j\rangle =\sum_{j=1}^{d}\alpha_{ji}|j\rangle$ is identified with the column vector ${\left(\alpha_{i1}^{*},\alpha_{i2}^{*},\cdots ,\alpha_{id}^{*}\right)}^{T}={\left(\alpha_{1i},\alpha_{2i},\cdots ,\alpha_{di}\right)}^{T}.$ Consequently, Ω can be expressed as the following Gram matrix

(24) $$\Omega =\left(\begin{array}{c} \langle \alpha_{1}|\\ \langle \alpha_{2}|\\ \vdots \\ \langle \alpha_{d}| \end{array}\right)(|\alpha_{1}\rangle ,|\alpha_{2}\rangle ,\cdots ,|\alpha_{d}\rangle )=\left(\langle \alpha_{i}|\alpha_{j}\rangle \right),$$

which will be used later.

We write the operation determined by Equation (22) as the channel

(25) $$\Phi_{S}\left(\rho_{S}\right)=\rho_{S}^{\prime }=\rho_{S}\circ \Omega ,$$

which may be called a Hadamard channel due to the involvement of the Hadamard product. This channel has some nice properties.

(a) The dual of the channel $\Phi_{S}\left(\rho_{S}\right)=\rho_{S}\circ \Omega$ is $\Phi_{S}^{†}\left(X\right)=X\circ \Omega^{T},$ where $\Omega^{T}$ denotes the transposition of the matrix Ω. In particular, if Ω is a real symmetric matrix and thus $\Omega^{T}=\Omega^{*}=\Omega ,$ then the corresponding channel $\Phi_{S}\left(\rho_{S}\right)$ is self-dual. Here $\Omega^{*}$ denotes complex conjugation of each entry of the matrix Ω.

(b) The channel $\Phi_{S}\left(\rho_{S}\right)=\rho_{S}\circ \Omega$ can be expressed as the Kraus operator-sum form

(26) $$\Phi_{S}\left(\rho_{S}\right)=\sum_{j=1}^{d}\Omega_{j}\rho_{S}\Omega_{j}^{†}$$

with the diagonal matrices (Kraus operators)

$$\Omega_{j}=\mathrm{diag}\left\{\alpha_{1j},\alpha_{2j},\cdots ,\alpha_{dj}\right\}=\left(\begin{array}{cccc} \alpha_{1j} & 0 & \cdots & 0\\ 0 & \alpha_{2j} & \cdots & 0\\ \vdots & \vdots & \ddots & \vdots \\ 0 & 0 & \cdots & \alpha_{dj} \end{array}\right)$$

with $\alpha_{ij}$ determined by Equation (23).

(c) If $|\omega_{ij}|<1$ for i≠j, then the repeated iteration of the channel $\Phi_{S}$ tends to the completely decohering channel in the sense that $\lim_{n\to \infty }\Phi_{S}^{n}\left(\rho_{S}\right)=\mathrm{diag}\left(\rho_{S}\right).$ Here the convergence is for any norm on the operator space of a finite dimensional Hilbert space.

All the above properties can be directly verified.

Compared with the initial system state $\rho_{S}$ given by Equation (19), the off-diagonal entries of the final system state $\rho_{S}^{\prime }=\Phi_{S}\left(\rho_{S}\right)$ in Equation (22) is multiplied by $\omega_{ij}$. This is the conventional meaning of decoherence as decaying of off-diagonal entries of the density matrix. In order to use a single numerical quantity to summarize certain amount of decoherence, we introduce the following quantity

(27) $$D\left(\rho_{S}|\Pi ,U\right)=J\left(\rho_{S}^{\prime },\Pi \right)-J\left(\rho_{S},\Pi \right),$$

which is our first key character for quantifying decoherence induced by the environment. The above quantifier of decoherence can be more explicitly expressed as

$$D\left(\rho_{S}|\Pi ,U\right)=\sum_{i=1}^{d}\left(\langle i|\sqrt{\rho_{S}^{\prime }}{|i\rangle }^{2}-\langle i|\sqrt{\rho_{S}}{|i\rangle }^{2}\right).$$

The physical intuition of the above quantity is the increase in classicality caused by the interaction with the environment (the channel $\Phi_{S}$). This notation indicates clearly and precisely that we are talking about the decoherence of the initial system state $\rho_{S}$ (with respect to Π) induced by the environment (symbolized by the collection $U=\{U_{E_{i}}:i=1,2,\cdots ,d\}$ of unitary operators acting on the environment).

The quantifier of decoherence $D(\rho_{S}|\Pi ,U)$ possesses the following properties.

**Proposition** **3.** *$0\leq D(\rho_{S}|\Pi ,U)\leq 1.$ Moreover, $D(\rho_{S}|\Pi ,U)=0$ if $U_{E_{i}}=c_{i}1_{E_{i}}$ (proportional to the identity operator on the i-th sub-environment for all i).*

*We conjecture that $D(\rho_{S}|\Pi ,U)$ is convex in ρ. It seems that a proof may require some deep mathematics.*

To prove Proposition 3, first noting that $\Phi_{S}^{†}\left(\rho_{S}\right)=\rho_{S}\circ \Omega^{T},$ it is easy to verify $\Phi_{S}^{†}\left(\Pi_{i}\right)=\Pi_{i}.$ Now, from $\Pi_{i}^{2}=\Pi_{i}$ and inequality (9) in Proposition 1, we conclude that

(28) $$J\left(\rho_{S}^{\prime },\Pi \right)=J\left(\Phi_{S}\left(\rho_{S}\right),\Pi \right)\leq J\left(\rho_{S},\Pi \right),$$

which implies the desired inequality $0\leq D(\rho_{S}|\Pi ,U).$ The upper bound $D(\rho_{S}|\Pi ,U)\leq 1$ follows readily from the property of J(ρ,Π).

For the decoherence channel $\Phi_{S}$, we have

(29) $$\lambda \left(\Phi_{S}\left(\rho_{S}\right)\right)≼\lambda \left(\rho_{S}\right),$$

where $\lambda (\rho_{S})$ is the probability vector consisting of the eigenvalues (spectrum, in any order) of the quantum state $\rho_{S}.$ The heuristic and intuitive meaning of the above inequality is that the decoherence renders the state flatter in the sense that the probability vector consisting of the eigenvalues of the final state is more uniform (more mixing, more spread out) than that of the initial system state, as mathematically defined by the majorization relation of probability vectors.

Recall that a unitarily invariant norm ||·|| is an operator norm with the unitary invariance ||X||=||UXW|| for all *X* and all unitary operators *U* and *W*. Prototypical examples of such norms include the trace norm, the Frobenius norm, the *p*-norm (with p≥1), and the Ky Fan norm [104]. Equation (29) implies that

(30) $$\left|\right|\Phi_{S}\left(\rho_{S}\right)\left|\right|\geq \left|\right|\rho_{S}\left|\right|$$

for any unitarily invariant norm ||·||. In particular, if $\Phi_{S}$ is the completely decohering channel in the sense that $\Omega =(\omega_{ij})=1,$ that is

$$\Phi_{S}\left(\rho_{S}\right)=\left(\begin{array}{cccc} \langle 1|\rho_{S}|1\rangle & 0 & \cdots & 0\\ 0 & \langle 2|\rho_{S}|2\rangle & \cdots & 0\\ \vdots & \vdots & \ddots & \vdots \\ 0 & 0 & \cdots & \langle d|\rho_{S}|d\rangle \end{array}\right)=\mathrm{diag}\left(\rho_{S}\right),$$

then we come to the well-known fact that the vector formed by the diagonal entries of a density matrix is majorized by the vector formed by the eigenvalues of the matrix [102], that is, $\lambda \left(\mathrm{diag}\left(\rho_{s}\right)\right)≼\lambda \left(\rho_{S}\right).$ By taking the trace norm of the logarithm of the states, we obtain $\Pi_{i=1}^{d}\langle i|\rho_{S}|i\rangle \geq \det\left(\rho_{S}\right)=\Pi_{i=1}^{d}\lambda_{i}\left(\rho_{S}\right),$ which is precisely the celebrated Hadamard determinant inequality [105]. Here $\lambda_{i}\left(\rho_{S}\right)$ are eigenvalues of $\rho_{S},$ and $\lambda \left(\rho_{S}\right)=\left(\lambda_{1}\left(\rho_{S}\right),\lambda_{2}\left(\rho_{S}\right),\cdots ,\lambda_{d}\left(\rho_{S}\right)\right).$

In terms of classicality defined via variance of states, we introduce an alternative quantifier for decoherence as

(31) $$F\left(\rho_{S}|\Pi ,U\right)=C\left(\rho_{S}^{\prime },\Pi \right)-C\left(\rho_{S},\Pi \right),$$

which can be explicitly expressed as

(32) $$F\left(\rho_{S}|\Pi ,U\right)=\mathrm{tr}\left(\rho_{S}^{2}\right)-\mathrm{tr}\left(\rho_{S}^{\prime 2}\right)=\sum_{i=1}^{d}\left(\langle i|\rho_{S}^{2}|i\rangle -\langle i|\rho_{S}^{\prime 2}|i\rangle \right).$$

The intuition of the above measure is similar to that of $D(\rho_{S}|\Pi ,U)$: The increase in classicality of the system caused by the environment captures some essential features of decoherence.

**Proposition** **4.**

*(a)*  *$0\leq F(\rho_{S}|\Pi ,U)\leq 1.$ Moreover, $F(\rho_{S}|\Pi ,U)=0$ if $U_{E_{i}}=c_{i}1_{E_{i}}$ (proportional to the identity operator on the i-th sub-environment for all i).*
*(b)*  *$F(\rho_{S}|\Pi ,U)$ is convex in ρ.*

For item (a), the non-negativity of $F(\rho_{S}|\Pi ,U)$ follows from inequality (16) in Proposition 2. The upper bound is evident in view of inequality (32).

For item (b), let c∈[0,1],$\rho_{S}$ and $\sigma_{S}$ be two states with $\rho_{S}^{\prime }=\Phi_{S}\left(\rho_{S}\right),\sigma_{S}^{\prime }=\Phi_{S}\left(\sigma_{S}\right).$ Straightforward manipulation yields

$$\begin{array}{cc} \; & cF\left(\rho_{S}|\Pi ,U\right)+\left(1-c\right)F\left(\sigma_{S}|\Pi ,U\right)-F\left(c\rho_{S}+\left(1-c\right)\sigma_{S}|\Pi ,U\right)\\ \; & =c\left(1-c\right)\left(\mathrm{tr}\left({\left(\rho_{S}-\sigma_{S}\right)}^{2}\right)-\mathrm{tr}\left({\left(\rho_{S}^{\prime }-\sigma_{S}^{\prime }\right)}^{2}\right)\right)\\ \; & \geq 0. \end{array}$$

The last inequality follows from

$$\lambda \left(\rho_{S}^{\prime }-\sigma_{S}^{\prime }\right))≼\lambda \left(\rho_{S}-\sigma_{S}\right).$$

We see that, on the one hand, $D(\rho_{S}|\Pi ,U)$ and $F(\rho_{S}|\Pi ,U)$ share some similar properties, and, on the other hand, they have different advantages and disadvantages. This is reminiscent of the comparison between the conventional variance and Fisher information.

## 4. Influence on Environment Caused by System

The interaction between the system and the environment is mutual. While we are focusing on the decoherence of the system caused by the environment, it may also be useful to investigate the influence on the environment caused by the system. In a formal fashion, this may also be interpreted as the decoherence of the environment caused by the system. Due to the asymmetry of the system–environment interaction, there are subtle differences between the influence on the environment caused by the system and that on the system caused by the environment.

From Equation (20), we obtain the final environment state

$$\rho_{E}^{\prime }={\mathrm{tr}}_{S}\rho_{SE}^{\prime }=\sum_{i=1}^{d}\mathrm{tr}\left(\Pi_{i}\rho_{S}\right)U_{E_{i}}\rho_{E}U_{E_{i}}^{†}=\sum_{i=1}^{d}p_{i}U_{E_{i}}\rho_{E}U_{E_{i}}^{†}$$

after the system–environment interaction. Here $p_{i}=\mathrm{tr}\left(\rho_{S}\Pi_{i}\right)=\langle i|\rho_{S}|i\rangle .$ We denote the above operation as

$$\Phi_{E}\left(\rho_{E}\right)=\rho_{E}^{\prime }=\sum_{i=1}^{d}p_{i}U_{E_{i}}\rho_{E}U_{E_{i}}^{†},$$

which is a random unitary channel with Kraus operators $\sqrt{p_{i}}U_{E_{i}}.$ Moreover, noting that $\rho_{E}=\rho_{E_{1}}⊗\rho_{E_{2}}⊗\cdots ⊗\rho_{E_{d}},$ we have

(33) $$\begin{array}{cc} J(\rho_{E},\Phi_{E}) & =1+\mathrm{tr}\left(\sqrt{\rho_{E}}\Phi_{E}^{†}\left(\sqrt{\rho_{E}}\right)\right)=1+\sum_{i=1}^{d}p_{i}\mathrm{tr}\left(\sqrt{\rho_{E}}U_{E_{i}}^{†}\sqrt{\rho_{E}}U_{E_{i}}\right)\\ \; & =1+\sum_{i=1}^{d}p_{i}\mathrm{tr}\left(\sqrt{\rho_{E_{i}}}U_{E_{i}}^{†}\sqrt{\rho_{E_{i}}}U_{E_{i}}\right) \end{array}$$

and

$$J\left(\rho_{E}^{\prime },\Phi_{E}\right)=1+\mathrm{tr}\left(\sqrt{\rho_{E}^{\prime }}\Phi_{E}^{†}\left(\sqrt{\rho_{E}^{\prime }}\right)\right)=1+\sum_{i=1}^{d}p_{i}\mathrm{tr}\left(\sqrt{\rho_{E}^{\prime }}U_{E_{i}}^{†}\sqrt{\rho_{E}^{\prime }}U_{E_{i}}\right)$$

The final state of the *i*-th sub-environment reads

$$\rho_{E_{i}}^{\prime }={\mathrm{tr}}_{{\hat{E}}_{i}}\rho_{E}^{\prime }={\mathrm{tr}}_{{\hat{E}}_{i}}\left(\sum_{i=1}^{d}p_{i}U_{E_{i}}\rho_{E}U_{E_{i}}^{†}\right)=p_{i}U_{E_{i}}\rho_{E_{i}}U_{E_{i}}^{†}+\left(1-p_{i}\right)\rho_{E_{i}},$$

where the notation ${\mathrm{tr}}_{{\hat{E}}_{i}}$ denotes the partial trace over all sub-environments except for $E_{i}$. We denote the corresponding operation as the channel

$$\Phi_{E_{i}}\left(\rho_{E_{i}}\right)=\rho_{E_{i}}^{\prime }=p_{i}U_{E_{i}}\rho_{E_{i}}U_{E_{i}}^{†}+\left(1-p_{i}\right)\rho_{E_{i}},$$

which is also a random unitary channel. The classicality of the environment can be evaluated as

(34) $$J\left(\rho_{E_{i}},\Phi_{E_{i}}\right)=1+\mathrm{tr}\left(\sqrt{\rho_{E_{i}}}\Phi_{E_{i}}^{†}\left(\sqrt{\rho_{E_{i}}}\right)\right)=2-p_{i}+p_{i}\mathrm{tr}\left(\sqrt{\rho_{E_{i}}}U_{E_{i}}^{†}\sqrt{\rho_{E_{i}}}U_{E_{i}}\right)$$

and

$$J\left(\rho_{E_{i}}^{\prime },\Phi_{E_{i}}\right)=1+\mathrm{tr}\left(\sqrt{\rho_{E_{i}}^{\prime }}\Phi_{E_{i}}^{†}\left(\sqrt{\rho_{E_{i}}^{\prime }}\right)\right)=2-p_{i}+p_{i}\mathrm{tr}\left(\sqrt{\rho_{E_{i}}^{\prime }}U_{E_{i}}^{†}\sqrt{\rho_{E_{i}}^{\prime }}U_{E_{i}}\right).$$

Comparing Equations (33) and (34), we get

$$2-J\left(\rho_{E},\Phi_{E}\right)=\sum_{i=1}^{d}\left(2-J(\rho_{E_{i}},\Phi_{E_{i}})\right),$$

or equivalently,

$$I\left(\rho_{E},\Phi_{E}\right)=\sum_{i=1}^{d}I\left(\rho_{E_{i}},\Phi_{E_{i}}\right),$$

which shows a kind of additivity property, as intuitively expected since the initial environment is in a product state $\rho_{E}=\rho_{E_{1}}⊗\rho_{E_{2}}⊗\cdots ⊗\rho_{E_{d}}.$

In terms of the classicality $J(\rho_{E},\Phi_{E})$ of the environment, we define the influence on the total environment caused by the system as

(35) $$\begin{array}{cc} D(\rho_{E}|U,\Pi ) & =J\left(\rho_{E}^{\prime },\Phi_{E}\right)-J\left(\rho_{E},\Phi_{E}\right) \end{array}$$

and the influence on the *i*-th sub-environment caused by the system as

(36) $$D\left(\rho_{E_{i}}|U,\Pi \right)=J\left(\rho_{E_{i}}^{\prime },\Phi_{E_{i}}\right)-J\left(\rho_{E_{i}},\Phi_{E_{i}}\right),$$

respectively. Compared with Equation (27), we have deliberately swapped the place of U and Π to indicate the difference of the reference channels. The influence on the environment caused by the system can be explicitly evaluated as

$$D\left(\rho_{E}|U,\Pi \right)=\sum_{i=1}^{d}p_{i}\mathrm{tr}\left(\sqrt{\rho_{E}^{\prime }}U_{E_{i}}^{†}\sqrt{\rho_{E}^{\prime }}U_{E_{i}}-\sqrt{\rho_{E}}U_{E_{i}}^{†}\sqrt{\rho_{E}}U_{E_{i}}\right).$$

Similarly,

$$D\left(\rho_{E_{i}}|U,\Pi \right)=p_{i}\mathrm{tr}\left(\sqrt{\rho_{E_{i}}^{\prime }}U_{E_{i}}^{†}\sqrt{\rho_{E_{i}}^{\prime }}U_{E_{i}}-\sqrt{\rho_{E_{i}}}U_{E_{i}}^{†}\sqrt{\rho_{E_{i}}}U_{E_{i}}\right).$$

If we use the alternative quantifier of classicality C(ρ,K), then the classicality of the initial and final environment states with respect to the reduced environment channel can be evaluated as

$$\begin{array}{cc} C(\rho_{E},\Phi_{E}) & =1-\mathrm{tr}\left(\rho_{E}^{2}\right)+\sum_{i=1}^{d}p_{i}^{2}\left(2-p_{i}d_{i}\right)+d-2\\ C(\rho_{E}^{\prime },\Phi_{E}) & =1-\mathrm{tr}\left(\rho_{E}^{\prime 2}\right)+\sum_{i=1}^{d}p_{i}^{2}\left(2-p_{i}d_{i}\right)+d-2. \end{array}$$

where $d_{i}$ is the dimension of the *i*-th sub-environment. In terms of the classicality of the environment $C(\rho_{E},\Phi_{E})$, we have an alternative measure of influence on the total environment caused by the system as

$$\begin{array}{cc} F(\rho_{E}|U,\Pi ) & =C\left(\rho_{E}^{\prime },\Phi_{E}\right)-C\left(\rho_{E},\Phi_{E}\right) \end{array}$$

and the influence on the *i*-th environment caused by the system as

$$\begin{array}{cc} F(\rho_{E_{i}}|U,\Pi ) & =C\left(\rho_{E_{i}}^{\prime },\Phi_{E_{i}}\right)-C\left(\rho_{E_{i}},\Phi_{E_{i}}\right), \end{array}$$

respectively. It turns out that

$$\begin{array}{cc} F(\rho_{E}|U,\Pi ) & =\mathrm{tr}\left(\rho_{E}^{2}\right)-\mathrm{tr}\left(\rho_{E}^{\prime 2}\right)\\ F(\rho_{E_{i}}|U,\Pi ) & =\mathrm{tr}\left(\rho_{E_{i}}^{2}\right)-\mathrm{tr}\left(\rho_{E_{i}}^{\prime 2}\right). \end{array}$$

These quantities of influence on the environment (caused by the system) may be compared with the quantifiers of decoherence of the system (caused by the environment), and they should be correlated due to the system-environment coupling.

## 5. Illustrating Decoherence in Interferometry

**Figure 2:**
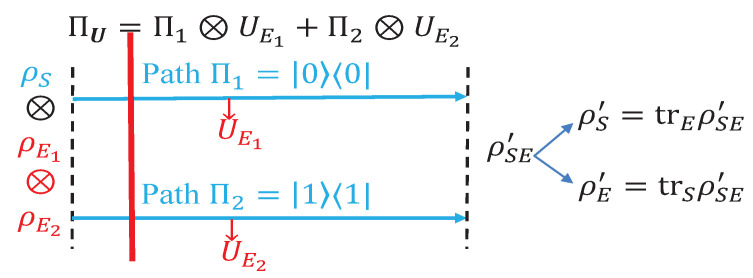
Schematic illustration of decoherence induced by the array of path detectors $U=\{U_{E_{i}}:i=1,2\}$ (serving as the environment of the system state $\rho_{S}$) attached to the collection of path $\Pi =\{\Pi_{i}:i=1,2\}$. Each path $\Pi_{i}$ is probed by a detector $U_{E_{i}}$. The initial system state is $\rho_{S}$, while the initial array of detector state is $\rho_{E}=\rho_{E_{1}}⊗\rho_{E_{2}}.$ The combined initial state is $\rho_{SE}=\rho_{S}⊗\rho_{E}.$ The system–detector coupling is via the combined unitary operator $\Pi_{U}=\sum_{i=1}^{2}\Pi_{i}⊗U_{E_{i}},$ and the final combined system is $\rho_{SE}^{\prime }=\Pi_{U}\rho_{SE}\Pi_{U}^{†}$ with final system state $\rho_{S}^{\prime }=\mathrm{tr}\rho_{SE}^{\prime }.$ The decoherence of $\rho_{S}$ (with respect to Π) induced by the path detectors are quantified by $D\left(\rho_{S}|\Pi ,U\right)=J\left(\rho_{S}^{\prime },\Pi \right)-J\left(\rho_{S},\Pi \right)$ and $F\left(\rho_{S}|\Pi ,U\right)=C\left(\rho_{S}^{\prime },\Pi \right)-C\left(\rho_{S},\Pi \right),$ which are the increasing amount of classicality of the system state caused by the path detectors.

We illustrate the effectiveness of the quantifiers proposed in the preceding section with a two-path interferometer, as depicted in Figure 2. The system Hilbert space of interest here is effectively a qubit space with the two paths labeled as $\Pi_{1}=\left|0\rangle \langle 0\right|$ and $\Pi_{2}=|1\rangle \langle 1|.$ Let the initial system state (the path degree part of the physical state) be

(37) $$\rho_{S}=\frac{1}{2}\left(1+\sum_{i=1}^{3}r_{j}\sigma_{j}\right)=\frac{1}{2}\left(\begin{array}{cc} 1+r_{3} & r_{1}-ir_{2}\\ r_{1}+ir_{2} & 1-r_{3} \end{array}\right)$$

with the Bloch vector $r=\left(r_{1},r_{2},r_{3}\right)\in R^{3}$ satisfying $\left|r\right|=\sqrt{r_{1}^{2}+r_{2}^{2}+r_{3}^{2}}\leq 1$ and $\sigma_{j}$ being the Pauli spin matrices. The eigenvalues of $\rho_{S}$ are (1±|r|)/2. It can be directly evaluated that

$$\sqrt{\rho_{S}}=\frac{1}{2\sqrt{\gamma }}\left(\begin{array}{cc} \gamma +r_{3} & r_{1}-ir_{2}\\ r_{1}+ir_{2} & \gamma -r_{3} \end{array}\right)$$

with

(38) $$\gamma =1+\sqrt{1-{\left|r\right|}^{2}}.$$

For a two-path interferometer with a detector attached to each path, let $\rho_{E_{i}}$ be the initial detector state attached to path i; the system and detector evolve under the controlled-U operation

(39) $$\Pi_{U}=\Pi_{1}⊗U_{E_{1}}+\Pi_{2}⊗U_{E_{2}}.$$

From an information-theoretic point of view, this controlled-U operation correlates the quantum system and the detector and leads to the combined final state

(40) $$\begin{array}{cc} \rho_{SE}^{\prime } & =\Pi_{U}\left(\rho_{S}⊗\rho_{E}\right)\Pi_{U}^{†}\\ \; & =\left(\Pi_{1}\rho_{S}\Pi_{1}\right)⊗\left(U_{E_{1}}\rho_{E}U_{E_{1}}^{†}\right)+\left(\Pi_{1}\rho_{S}\Pi_{2}\right)⊗\left(U_{E_{1}}\rho_{E}U_{E_{2}}^{†}\right)\\ \; & \;+\left(\Pi_{2}\rho_{S}\Pi_{1}\right)⊗\left(U_{E_{2}}\rho_{E}U_{E_{1}}^{†}\right)+\left(\Pi_{2}\rho_{S}\Pi_{2}\right)⊗\left(U_{E_{2}}\rho_{E}U_{E_{2}}^{†}\right). \end{array}$$

The final system state can be obtained by taking the partial trace over the detector as

$$\begin{array}{cc} \rho_{S}^{\prime } & ={\mathrm{tr}}_{E}\rho_{SE}^{\prime }\\ \; & =\Pi_{1}\rho_{S}\Pi_{1}+\Pi_{1}\rho_{S}\Pi_{2}\mathrm{tr}\left(U_{E_{1}}\rho_{E}U_{E_{2}}^{†}\right)+\Pi_{2}\rho_{S}\Pi_{1}\mathrm{tr}\left(U_{E_{2}}\rho_{E}U_{E_{1}}^{†}\right)+\Pi_{2}\rho_{S}\Pi_{2}\\ \; & =\frac{1}{2}\left(\begin{array}{cc} 1+r_{3} & \left(r_{1}-ir_{2}\right)V^{*}\\ (r_{1}+ir_{2})V & 1-r_{3} \end{array}\right). \end{array}$$

with

$$V=\mathrm{tr}\left(U_{E_{2}}\rho_{E}U_{E_{1}}^{†}\right)=\mathrm{tr}\left(U_{E_{2}}\rho_{E_{2}}\right)\cdot \mathrm{tr}\left(\rho_{E_{1}}U_{E_{1}}^{†}\right)$$

being a complex number. By the Cauchy–Schwarz inequality, we have

$$\begin{array}{cc} {\left|V\right|}^{2} & =|\mathrm{tr}\left(\left(U_{E_{2}}\sqrt{\rho_{E}}\right){\left(U_{E_{1}}\sqrt{\rho_{E}}\right)}^{†}\right){|}^{2}\\ \; & \leq \mathrm{tr}\left(\left(U_{E_{2}}\sqrt{\rho_{E}}\right){\left(U_{E_{2}}\sqrt{\rho_{E}}\right)}^{†}\right)\cdot \mathrm{tr}\left(\left(U_{E_{1}}\sqrt{\rho_{E}}\right){\left(U_{E_{1}}\sqrt{\rho_{E}}\right)}^{†}\right)\\ \; & =1. \end{array}$$

The eigenvalues of $\rho_{S}^{\prime }=\Phi_{S}\left(\rho_{S}\right)$ are

(41) $$\begin{array}{cc} \lambda_{1}\left(\rho_{S}^{\prime }\right) & =\frac{1}{2}\left(1+\sqrt{r_{3}^{2}+\left(r_{1}^{2}+r_{2}^{2}\right){\left|V\right|}^{2})}\right) \end{array}$$

(42) $$\begin{array}{cc} \lambda_{2}\left(\rho_{S}^{\prime }\right) & =\frac{1}{2}\left(1-\sqrt{r_{3}^{2}+\left(r_{1}^{2}+r_{2}^{2}\right){\left|V\right|}^{2}}\right). \end{array}$$

Consequently, we see that

$$\lambda \left(\rho_{S}^{\prime }\right)≼\lambda \left(\rho_{S}\right),$$

as it should be by Proposition 2.

Noting that

$$\sqrt{\rho_{S}^{\prime }}=\frac{1}{2\sqrt{\gamma^{\prime }}}\left(\begin{array}{cc} \gamma^{\prime }+r_{3} & \left(r_{1}-ir_{2}\right)V^{*}\\ (r_{1}+ir_{2})V & \gamma^{\prime }-r_{3} \end{array}\right),$$

where

(43) $$\gamma^{\prime }=1+\sqrt{1-\left(r_{1}^{2}+r_{2}^{2}\right){\left|V\right|}^{2}-r_{3}^{2}},$$

we obtain

(44) $$\begin{array}{cc} D(\rho_{S}|\Pi ,U) & =J\left(\rho_{S}^{\prime },\Pi \right)-J\left(\rho_{S},\Pi \right)\\ \; & ={\left(\frac{\gamma^{\prime }+r_{3}}{2\sqrt{\gamma^{\prime }}}\right)}^{2}+{\left(\frac{\gamma^{\prime }-r_{3}}{2\sqrt{\gamma^{\prime }}}\right)}^{2}-{\left(\frac{\gamma +r_{3}}{2\sqrt{\gamma }}\right)}^{2}-{\left(\frac{\gamma -r_{3}}{2\sqrt{\gamma }}\right)}^{2}\\ \; & =\frac{1}{2}\left(\gamma^{\prime }-\gamma \right)\left(1-\frac{r_{3}^{2}}{\gamma^{\prime }\gamma }\right). \end{array}$$

Since $\gamma^{\prime }\geq \gamma ,\gamma \geq 1\geq r_{3},$ we see readily that the above quantity is non-negative. Moreover, because $\gamma^{\prime }$ is a decreasing function of ${\left|V\right|}^{2},$ from Equation (44) we see that $D(\rho_{S}|\Pi ,U)$ is a decreasing function of ${\left|V\right|}^{2}.$

Similarly, we can evaluate

$$\begin{array}{cc} C(\rho_{S}^{\prime },\Pi ) & =1-\frac{1}{4}\left({\left(1+r_{3}\right)}^{2}+\left(r_{1}^{2}+r_{2}^{2}\right){\left|V\right|}^{2}+{\left(1-r_{3}\right)}^{2}+\left(r_{1}^{2}+r_{2}^{2}\right){\left|V\right|}^{2}\right)\\ \; & \;+\frac{1}{4}\left({\left(1+r_{3}\right)}^{2}+{\left(1-r_{3}\right)}^{2}\right)\\ \; & =1-\frac{1}{2}\left(r_{1}^{2}+r_{2}^{2}\right){\left|V\right|}^{2}, \end{array}$$

from which we obtain

(45) $$F\left(\rho_{S}|\Pi ,U\right)=C\left(\rho_{S}^{\prime },\Pi \right)-C\left(\rho_{S},\Pi \right)=\frac{1}{2}\left(r_{1}^{2}+r_{2}^{2}\right){\left(1-|V\right|}^{2}),$$

which is also apparently a decreasing function of |V|.

It can be easily verified that both the decoherence quantifiers $D(\rho_{S}|\Pi ,U)$ and $F(\rho_{S}|\Pi ,U)$ are decreasing functions of |V|, and achieve the minimal value 0 when |V|=1, which corresponds to the situation when the detector does not obtain the path information. In this case, coherence is preserved, and there is no decoherence. This is consistent with our intuition since decoherence can be regarded as the washing out of interference, while $D(\rho_{S}|\Pi ,U)$ and $F(\rho_{S}|\Pi ,U)$ can be regarded as measures of path information leakage to the detectors (classical path information). The detectors, from which we can obtain the path information of the quantum system, would inevitably reduce the interference ability of the quantum system.

The quantity $V=\mathrm{tr}(U_{E_{2}}\rho_{E}U_{E_{1}}^{†})$ arises naturally in at least two other contexts:

(a) If we take $U_{E_{1}}=1$ and $U_{E_{2}}=U$, then we come to the setup of Englert [34], in which |V| is the fringe visibility in the complementarity relation

$${\left|V\right|}^{2}+D^{2}\leq 1,$$

with $D=\frac{1}{2}\mathrm{tr}\left|U\rho_{E}U^{†}-\rho_{E}\right|$ being the quantitative measure of distinguishability. In this context, V is also called the interference function.

(b) If we define the generalized variance of measuring any operator *X* in state σ as

$$V\left(\sigma ,X\right)=\mathrm{tr}\left(\sigma \left(X-\mathrm{tr}\left(\sigma X\right)\right){\left(X-\mathrm{tr}\left(\sigma X\right)\right)}^{†}\right),$$

and consider the unitary operator $U_{E_{1}}^{†}U_{E_{2}}=U_{E_{1}}^{†}⊗U_{E_{2}},$ then we have

$$\begin{array}{cc} V(\rho_{E},U_{E_{1}}^{†}U_{E_{2}}) & =\mathrm{tr}\left(\rho_{E}\left(U_{E_{1}}^{†}U_{E_{2}}-\mathrm{tr}\left(\rho_{E}U_{E_{1}}^{†}U_{E_{2}}\right)\right){\left(U_{E_{1}}^{†}U_{E_{2}}-\mathrm{tr}\left(\rho_{E}U_{E_{1}}^{†}U_{E_{2}}\right)\right)}^{†}\right)\\ & =1-|\mathrm{tr}\left(U_{E_{2}}\rho_{E}U_{E_{1}}^{†}\right){|}^{2}\\ \; & =1-{\left|V\right|}^{2}, \end{array}$$

which is a kind of measure of path detecting capability. The above relation immediately leads to

$$\begin{array}{c} V\left(\rho_{E},U_{E_{1}}^{†}U_{E_{2}}\right)+{\left|V\right|}^{2}=1, \end{array}$$

which is apparently a complementary relation between the path information and fringe visibility. Furthermore, combined with Equation (45), we have

$$F\left(\rho_{S}|\Pi ,U\right)=\frac{1}{2}\left(r_{1}^{2}+r_{2}^{2}\right)V\left(\rho_{E},U_{E_{1}}^{†}U_{E_{2}}\right),$$

which relates the decoherence directly with the path-detecting information. This is consistent with our intuitive understanding of decoherence as the information leakage to the detectors (environment).

Now we make some comparison of our quantifiers of decoherence with existing ones. Since, in general, decoherence is also regarded as the establishment of correlations between the system and environment, it is expected that decoherence should be related to correlations, as quantified by the mutual information between the system and the environment. For simplicity, we consider the setup described by Figure 2 and assume that the initial system state and environment state are both pure. In this case, the final system–environment state $\rho_{SE}^{\prime }$ is pure since the coupling $\Pi_{U}$ is unitary. Consequently, the mutual information of the final system–environment state is

$$I\left(\rho_{SE}^{\prime }\right)=S\left(\rho_{S}^{\prime }\right)+S\left(\rho_{E}^{\prime }\right)-S\left(\rho_{SE}^{\prime }\right)=2S\left(\rho_{S}^{\prime }\right)=2\left(-\lambda_{1}\left(\rho_{S}^{\prime }\right)\ln\lambda_{1}\left(\rho_{S}^{\prime }\right)-\lambda_{2}\left(\rho_{S}^{\prime }\right)\ln\lambda_{2}\left(\rho_{S}^{\prime }\right)\right),$$

where $\lambda_{j}\left(\rho_{S}^{\prime }\right)$ are determined by Equations (41) and (42), and S(σ)=−trσlnσ is the von Neumann entropy of the state σ. Since the initial system state $\rho_{S}$ defined by Equation (37) is pure, we have $r_{1}^{2}+r_{2}^{2}+r_{3}^{2}=1.$ Therefore by Equations (41), (42) and (45), we have

$$\begin{array}{cc} \lambda_{1}\left(\rho_{S}^{\prime }\right) & =\frac{1}{2}\left(1+\sqrt{1-\left(r_{1}^{2}+r_{2}^{2}\right){\left(1-|V\right|}^{2})}\right)=\frac{1}{2}\left(1+\sqrt{1-2F(\rho_{S}|\Pi ,U)}\right),\\ \lambda_{2}\left(\rho_{S}^{\prime }\right) & =\frac{1}{2}\left(1-\sqrt{1-\left(r_{1}^{2}+r_{2}^{2}\right){\left(1-|V\right|}^{2})}\right)=\frac{1}{2}\left(1-\sqrt{1-2F(\rho_{S}|\Pi ,U)}\right). \end{array}$$

Now the mutual information can be expressed as

$$I\left(\rho_{SE}^{\prime }\right)=2H\left(\frac{1}{2}\left(1+\sqrt{1-2F(\rho_{S}|\Pi ,U)}\right)\right),$$

where H(p)=−plnp−(1−p)ln(1−p) is the binary Shannon entropy function, 0≤p≤1. From the above equation, we see that the mutual information is monotonically related to the decoherence: when decoherence increases, the mutual information increases, which is consistent with the intuition that larger decoherence corresponds to larger amount of correlations established between the system and the environment (larger information leakage to the environment). Although the above result is proved for initial pure states and $F(\rho_{S}|\Pi ,U)$, the general cases concerning mixed initial states and the decoherence quantifier $D(\rho_{S}|\Pi ,U)$ are similar, but the calculations are more complicated. It will be also interesting to make a more comprehensive comparative studies between various quantities related to decoherence and correlations.

## 6. Summary

In order to quantify decoherence induced by environment, we reviewed two quantifiers of classicality in a general setup of state–channel interaction by exploiting the Jordan symmetric product and a modified notion of variance. These quantifiers may be of independent interest in addressing the classical–quantum interplay. We also elucidated some simple yet useful features of the decoherence channel (Hadamard channel).

Employing the above quantifiers of classicality, we introduced two quantifiers of decoherence induced by environment in the combined “system + environment” setup. These quantifiers have some nice properties and can be used to summarize the decoherence strength of an open system. Connections with complementarity were discussed. The results were illustrated via a two-path interferometer.

A natural approach to quantifying decoherence is via correlations between the system and the environment. There are various quantifiers for correlations such as the quantum mutual information, entanglement, quantum discord, measurement-induced disturbance, measurement-induced nonlocality, classical correlations, etc. In particular, decoherence is quantified from a decorrelating perspective in Refs. [106,107]. However, correlations are generally hard to evaluate. Our present approaches differ from the conventional approach to decoherence via correlations such as quantum mutual information. Our quantifiers of decoherence are relatively easier to calculate and have intimate relations with the Wigner–Yanase skew information, uncertainty, and the resource theory of coherence. This indicates certain operational significance of the quantities. However, it remains to further study the operational meaning of these quantifiers of decoherence and to investigate their implications for foundational issues and experimental practices.

For open quantum systems, apart from decoherence, another prominent characteristic is quantum Markovianity/non-Markovianity [108,109,110,111,112,113,114,115,116]. Although the classical Markovianity is uniquely defined and well understood, there is not a single universally accepted definition of quantum Markovianity. A host of quantum Markovianity-related concepts coexist, such as GKS–Lindblad master equations, distinguishability, divisibility, no-information backflow, monotonic decreasing in correlations, etc. However, just like decoherence is related to the decaying of off-diagonal entries of the density matrix, a general common feature of the various Markovianities is related to information loss and memoryless effects. This indicates that there are intimate relations between decoherence and Markovianity. We remark that the feature of decoherence as information monotonically flowing into the environment is deeply related to the Markovian approximation. In non-Markovian dynamics, in contrast to decoherence, recoherence may occur. The interplay and relations between decoherence and quantum Markovianity/non-Markovianity are worth further investigations.

## Data Availability

Not appliable.
